# Supplementary material for: Metagenomic binning with assembly graph embeddings
Source: Bioinformatics. 2022 Aug 16;38(19):4481–7. doi: 10.1093/bioinformatics/btac557 (PMC9525014; doi:10.1093/bioinformatics/btac557)
Supplement: btac557_supplementary_data [file btac557_supplementary_data.zip › assembly_graph_bin_supmaterial_25052022.pdf]

Supplementary Material for "Metagenomic binning with assembly graph embeddings"

May 25, 2022

1 Strong100 dataset description.

The strong100 dataset is a simulated dataset of long-read with a total of 100 strains from 45 species, where 20 species were represented by a single strain, and the others by multiple strains. We used the same species from [1] and the same genomes whenever possible. The full list of genomes used is provided as supplementary material. We downloaded those genomes and generated random coverages y_s for each species s based on a log-normal distribution, and normalized so that the sum of all y_s was 1. The number of bases simulated for each genome g belonging to species s was given by

$$bp_{s,g} = y_s \cdot p_g \cdot N$$

where p_g is sampled from a Dirichlet distribution according to the number of strains and N is the total number of bases to simulated, which we set to 7.5Gbp. We then use badread[1] (v0.2.0) to simulate long-reads of each genome. We set the mean read length to 10000 and standard deviation to 7000, mean, max and stdev identity to 98, 99.9 and 5, respectively, and the error model to the default `nanopore2020 model`. The simulated reads were then assembled with flye in the same way as the reads from real datasets. We provide the code to generate random reads at `https://github.com/AndreLamurias/binning_workflows`.

2 Real-world datasets

Table 1 shows the accession number and reference of the datasets used in this study.

3 Medium-Quality bins

Table 2 shows the number of MQ bins (>50 completeness and <10 contamination) obtained with each approach, and in comparison to GraphMB.

[1] `https://github.com/rrwick/Badread`

Table 1: Real-world datasets used in this study.

Name	Accession No.	Reference
Hjor	SRX8234968, SRX8234918, SRX8234919, SRX8234920	[2]
Viby	SRX8234979, SRX8234951, SRX8234952, SRX8234953	[2]
Damh	SRX8234959, SRX8234889, SRX8234890, SRX8234891	[2]
Mari	SRX8234971, SRX8234928, SRX8234929, SRX8234930	[2]
AalE	SRX8234954, SRX8234897, SRX8234912, SRX8234913	[2]
Hade	SRX8234965, SRX8234909, SRX8234910, SRX8234911	[2]
Soil	PRJEB50688	[3]

Table 2: Medium-quality (MQ) bins obtained with GraphMB and state-of-the-art binning tools.

HQ bins	Strong100	Hjor	Viby	Damh	Mari	AalE	Hade	Soil
GraphBin	31	78	93	158	110	123	158	63
Maxbin2	30	41		75	61	49	76	17
SemiBin-ocean	35	53	52	84	84	85	89	5
SemiBin-train	35	38	58	94	91	88	99	0
VAMB	31	79	77	131	126	151	152	2
MetaBAT2	38	96	110	204	144	167	206	63
**GraphMB**	33	75	109	191	153	181	232	51
Δ VAMB	2	-4	32	60	27	30	80	51
Δ MetaBAT	-5	-21	-1	-13	9	14	26	-12
Δ % VAMB	1.1%	-5.3%	29.4%	31.4%	17.6%	16.6%	34.5%	100.0%
Δ % MetaBAT	-2.8%	-28.0%	-0.9%	-6.8%	5.9%	7.7%	11.2%	-23.5%

4 Processing time and memory usage

Table 3 shows the processing time required for running VAMB to generate the initial embeddings, and running GraphMB to generate the final binning. The peak memory usage and number of edges is also reported.

Table 3: Running time and peak memory usage of GraphMB.

	VAMB time (s)	GraphMB time (s)	Peak Memory (MB)	# Edges
Strong100	107	64	79	670
Hjor	375	312	503	5937
Viby	554	288	635	7800
DamH	639	442	929	14066
Mari	643	442	979	12651
AalE	713	520	1002	12425
Hade	1211	897	1895	27952
Soil	1203	2872	8591	69522

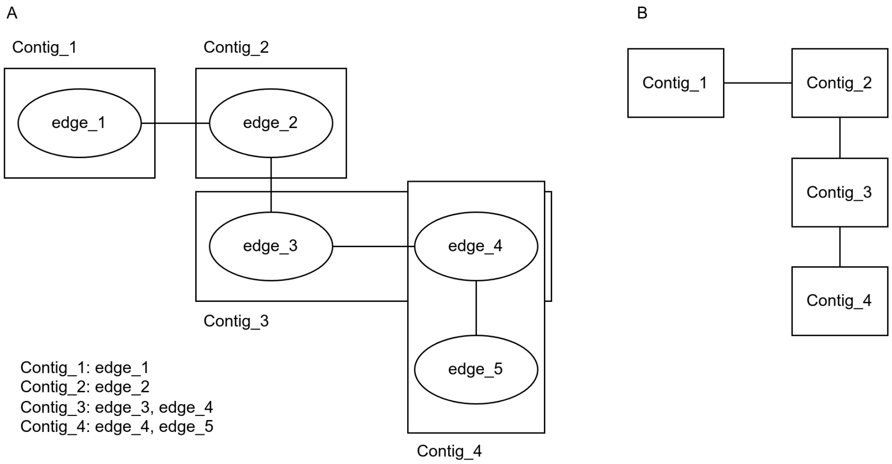

Figure 1: Diagram showing how contigs are mapped to the assembly graph. In this work, we use the left version of the graph, considering only the edge sequences. A simplified version of the graph is also shown on the right, where the contigs are connected according to their edge paths.

5 Short-read comparison

Table 4: Results on simHC short read dataset. AP - Average Purity, AC - Average contamination, both calculated with AMBER. Edges refers to using the edge sequences and contig using the contig sequences (which may contain multiple edges, see Supplementary Figure 1.

Method	Sequence	AP (bp)	AC (bp)	F1	HQ	MQ
VAMB	edges	0.986	0.808	0.888	25	63
VAMB	contigs	0.973	0.95	0.961	27	33
MetaBAT	edges	0.979	0.622	0.761	18	69
MetaBAT	contigs	0.988	0.646	0.781	30	64
GraphMB	edges	0.993	0.687	0.812	34	65

References

[1] Christopher Quince, Sergey Nurk, Sebastien Raguideau, Robert S James, Orkun S Soyer, J Kimberley Summers, Antoine Limasset, A Murat Eren, Rayan Chikhi, and Aaron E Darling. Metagenomics strain resolution on assembly graphs. *BioRxiv*, 2020.

[2] Caitlin M Singleton, Francesca Petriglieri, Jannie M Kristensen, Rasmus H Kirkegaard, Thomas Y Michaelsen, Martin H Andersen, Zivile Kondrotaite, Søren M Karst, Morten S Dueholm, Per H Nielsen, et al. Connecting structure to function with the recovery of over 1000

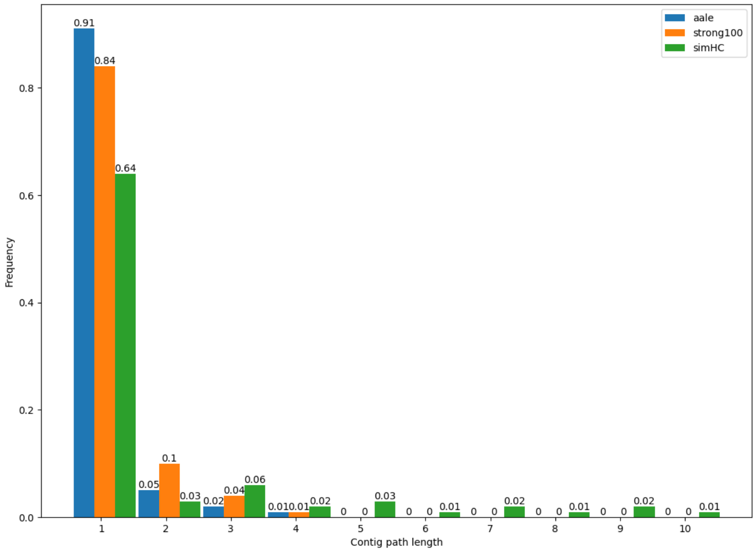

Figure 2: Frequency of contig path lengths for two long read datasets (AalE and Strong100) and one short-read dataset (simHC).

high-quality metagenome-assembled genomes from activated sludge using long-read sequencing. *Nature communications*, 12(1):1–13, 2021.

[3] Ane Kirstine Brunbjerg, Hans Henrik Bruun, Lars Brøndum, Aimée T Classen, Lars Dalby, Kåre Fog, Tobias G Frøslev, Irina Goldberg, Anders Johannes Hansen, Morten DD Hansen, et al. A systematic survey of regional multi-taxon biodiversity: evaluating strategies and coverage. *BMC ecology*, 19(1):1–15, 2019.
